# Validation of the PSMA PRIMARY Scoring System and Comparison to an E-PSMA Likert System for [^68^Ga]Ga-PSMA-11 PET/CT Interpretation in Men With Suspected Radiorecurrent Prostate Cancer

**DOI:** 10.1097/RLU.0000000000006168

**Published:** 2025-11-27

**Authors:** Alexander Light, Stefan Lazic, Martin J. Connor, Henry Tam, Hashim U. Ahmed, Taimur T. Shah, Tara D. Barwick

**Affiliations:** * Imperial Prostate, Department of Surgery and Cancer, Imperial College London; † Imperial Urology, Charing Cross Hospital, Imperial College Healthcare NHS Trust; ‡ Department of Imaging, Charing Cross Hospital, Imperial College Healthcare NHS Trust; § Department of Surgery and Cancer, Imperial College London, London, UK

**Keywords:** PSMA PET/CT, prostate cancer, PRIMARY, radiotherapy, recurrence, Likert

## Abstract

**Background:**

To validate the recently published 5-point PRIMARY scoring system against a 5-point European Association of Nuclear Medicine (E-PSMA) Likert system for the detection of intraprostatic recurrence for patients undergoing [^68^Ga]Ga-PSMA-11 PET/CT who have previously received radiotherapy for prostate cancer as their primary treatment.

**Patients and Methods:**

Thirty-five patients from one centre were investigated for suspected radiorecurrence between 2016 and 2022. All patients underwent multiparametric MRI and [^68^Ga]Ga-PSMA-11 PET/CT for staging, then prostate biopsy. Analyses were performed at the hemi-gland level. Two expert readers, blinded to other clinical, pathologic, and radiologic data, independently assigned each hemi-gland an E-PSMA Likert and PRIMARY score. Outcomes were comparative diagnostic accuracy metrics and interreader agreement for each system at the hemi-gland level. Scores of 3–5 were considered suspicious for intraprostatic cancer.

**Results:**

Of 70 hemi-glands, 43 (61%) had cancer on biopsy. Area under the curve was high and not statistically significantly different between systems (E-PSMA Likert: 0.84; 95% CI: 0.74–0.92; PRIMARY: 0.82; 95% CI: 0.71–0.91; *P* = 0.7). Sensitivity for E-PSMA Likert was 0.89 (95% CI: 0.78–0.96), not statistically different compared with the PRIMARY system (0.79; 95% CI: 0.67–0.89; *P* = 0.3). Specificity for the E-PSMA Likert system was 0.67 (95% CI: 0.46–0.86), not statistically different compared with the PRIMARY system (0.78; 95% CI: 0.60–0.91; *P* = 0.1). There was substantial interreader agreement between each reader for both E-PSMA Likert (κ = 0.65; 95% CI: 0.44–0.83) and PRIMARY systems (κ = 0.74; 95% CI: 0.59–0.90).

**Conclusions:**

In patients with biochemical recurrence after primary radiotherapy for prostate cancer, both the 5-point PRIMARY and E-PSMA Likert scoring systems score demonstrated good performance and substantial interreader agreement between experts for the detection of intraprostatic recurrences.

Prostate cancer is the most common male cancer, with radiotherapy being a first-line treatment for localized disease. However, within 10 years of radiotherapy, a quarter of patients will develop biochemical failure, a rise in serum prostate-specific antigen (PSA) highly suggestive of disease recurrence.^1^ Based on PSMA PET imaging, over 1 in 3 will have a localized recurrence that is confined to the prostate, a state independently predictive of subsequent metastasis and cancer-specific mortality.^2,3^ Salvage treatment directed to localized failures could improve upon these outcomes, a concept that has been demonstrated for various focal and whole gland salvage treatments.^4–11^

For patients who reach biochemical failure, both American and European guidelines recommend performing prostate MRI with whole-body PSMA PET/CT in those fit for salvage treatments.^12,13^ The principal objectives are to rule out metastatic disease while also identifying any suspicious intraprostatic lesions that warrant biopsy, a prerequisite for salvage treatment. The need for accurate diagnosis, localization, and characterization of all intraprostatic disease is pertinent for those considering salvage focal ablative treatments, which target the malignant lesion(s) alone rather than the whole prostate. MRI with MRI-targeted biopsy has been shown to have a high sensitivity for the detection of disease, but systematic biopsies are still recommended to capture MRI-inconspicuous disease elsewhere in the prostate.^7,14^

Although it has high specificity for identifying extra-prostatic spread, the role of PSMA PET/CT for identifying intraprostatic disease is less clear. Few studies have examined the ability of PSMA PET/CT to detect localized failures against a biopsy reference standard.^15,16^ The limited evidence available does suggest PSMA PET/CT has high sensitivity, particularly in conjunction with MRI.^17–21^ However, these studies are inconsistent in what determines a “suspicious” PET/CT, and there currently exists no validated scoring system in this setting. Our group has previously used a 5-point Likert system, aligning with European Association of Nuclear Medicine guidance (E-PSMA Likert).^21,22^ The Australian multicentre PRIMARY trial concluded that [^68^Ga]Ga-PSMA-11 PET/CT with MRI improved diagnosis of clinically-significant cancer compared with MRI alone in the primary diagnostic setting.^23^ Since then, the authors have developed and validated a 5-point PRIMARY PSMA PET/CT scoring system based on uptake pattern and intensity (Fig. 1), shown to have high sensitivity and substantial interreader agreement in the untreated prostate.^24,25^ This score has since been incorporated into the PROMISE v2 framework for local disease assessment.^26^ It is possible that this system could have comparable performance in irradiated prostates and could provide value in both standardizing and optimizing [^68^Ga]Ga-PSMA-11 PET/CT interpretation.

**FIGURE 1:**
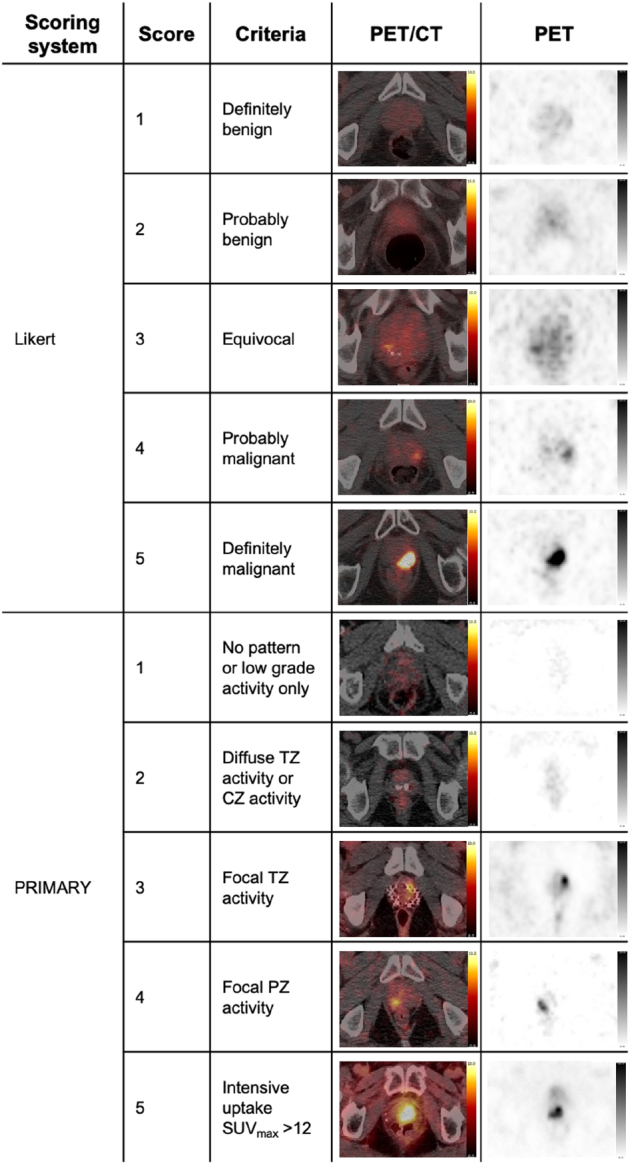
Comparison of the 5-point E-PSMA Likert and PRIMARY scoring systems with examples. CZ indicates central zone; PZ, peripheral zone; SUV_max_, maximum standardized uptake value; TZ, transitional zone.

To our knowledge, no study has evaluated the PRIMARY score in the radiorecurrent setting. The aim of this study was, therefore, to evaluate the diagnostic accuracy and interreader agreement of the PSMA PRIMARY system in the radiorecurrence setting and compare it to the E-PSMA Likert system.

## PATIENTS AND METHODS

### Study Design

This was a retrospective single-centre series for which local approval was received (registration number: URO_23). The study population comprised patients with prostate cancer undergoing [^68^Ga]Ga-PSMA-11 PET/CT with prostate biopsy at our UK centre for suspected recurrence after previous radiotherapy, either external-beam radiotherapy and/or high-dose or low-dose rate brachytherapy. Use of neoadjuvant and/or adjuvant androgen deprivation therapy was permitted. All patients had a rising PSA from nadir but no restrictions, for example the Phoenix criteria, were placed on specific PSA thresholds before referral for PET/CT.^27^ Local salvage treatment before PET/CT was an exclusion criterion. All patients also underwent a prostate MRI for evaluation of intraprostatic disease; no patient included had extra-prostatic disease identified either on PET/CT or prostate MRI. The diagnostic accuracy of prostate MRI in this same cohort has been previously published.^21^ At our centre, all patients with organ-confined radiorecurrent prostate cancer on imaging who were fit and willing for salvage focal therapy were offered a biopsy to identify, characterize, and map any intraprostatic disease in keeping with American and European guidelines.^13,28^ Four of the 35 included patients had a negative PET/CT and underwent biopsy for a suspicious MRI and/or persistently raised PSA with negative MRI.

### [^68^Ga]Ga-PSMA-11 PET/CT

All PET/CT examinations were performed on Siemens Healthineers Biograph 64, Biograph 128, or Biograph Vision scanners. Examinations included a standard knees-to-vertex acquisition, followed by a delayed postmicturition pelvic acquisition. Further details regarding this imaging protocol have been previously described.^21^

Images were reviewed independently by 2 expert readers (H.T. and T.D.B.), who were blinded to clinical, pathologic, and other radiologic data, including any accompanying prostate MRI. The prostate was divided into hemi-glands (left/right), and the suspicion of radiorecurrent disease ascertained for each hemi-gland. For each hemi-gland, each reader recorded a 5-point E-PSMA Likert score in 1 reporting session, then at a later time point recorded a 5-point PRIMARY score in a second reporting session using the same order of patients.^24^ The reader was also blinded to the previous scores given in the first reporting session. The use of different time points and blinding minimized the chance of a reader assigning a similar E-PSMA Likert and PRIMARY score that might occur if the reader recorded both scores in the same reporting session. Figure 1 outlines the criteria for each interpretation system with illustrative examples. A score of 3–5 for either system was deemed suspicious in this analysis, with analyses then repeated using a more stringent threshold of 4–5. If there was a discrepancy in reader scores for a given examination, the higher score was chosen as the score used in diagnostic accuracy analyses.

### Biopsy

All included patients underwent prostate biopsy of both prostate hemi-glands with image-targeted and/or systematic cores, including bilateral sampling of the peripheral zone as minimum that was sufficient for focal therapy planning. Targeted biopsies were performed cognitively. Of the patients, 30/35 (86%) underwent both systematic and targeted biopsy, 4/11 (11%) underwent systematic biopsy only, and 1/35 (3%) underwent targeted biopsy only.

### Outcomes

Analyses were performed primarily at the prostate hemi-gland level using cluster bootstrapping, with any cancer that crossed hemi-gland boundaries deemed as a separate cancer in each hemi-gland. The study outcomes were the diagnostic accuracy and the interreader agreement of the PRIMARY and E-PSMA Likert systems.

This analysis focused principally on the detection of “any cancer,” that is, cancer of any grade group and maximum cancer core length (MCCL). This is because the prognostic significance of tumor grade and size is less clear in the radiorecurrent setting. Here we also performed analyses with the following thresholds of disease that have been applied in primary diagnostic studies: (1) grade group ≥3 and/or MCCL ≥6 mm (PROMIS definition 1), and (2) grade group ≥2 and/or MCCL ≥4 mm (PROMIS definition 2).^29^

For the 2 PROMIS definitions, patients were excluded from calculations if a grade group or MCCL could not be given by the reporting pathologist. For example, if a patient had cancer with MCCL 2 mm but undeterminable grade due to irradiation effect, then the patient would be excluded from definition 1 and 2 calculations. However, if they had MCCL 6 mm, they would be considered as having both definition 1 and 2 cancer, despite grade being undeterminable.

### Statistical Analysis

At the hemi-gland level, because 2 hemi-glands from 1 patient are inherently nonindependent, diagnostic accuracy metrics were calculated using cluster bootstrapping with 1000 resamples, with each patient representing a cluster. This was performed to generate 95% CI. Smoothed bootstrapped receiver operating characteristic (ROC) curves were generated for each scoring system, with area under the curve (AUC) values compared using the DeLong test.^30,31^ The McNemar test was used to compare the sensitivity and specificity of the systems, with these 2 metrics tested discretely in diseased and nondiseased populations, respectively.^32^ A general estimating equation (GEE) logistic regression model was used to compare the positive predictive value (PPV) and negative predictive value (NPV) of each scoring system.^33^

Analyses were performed first using a score threshold of 3–5 to indicate a suspicious examination, then increased to a threshold of 4–5. Analyses were also performed at the whole gland level.

Interreader agreement was calculated using Cohen kappa (κ).^34^ κ was interpreted using the following thresholds: <0: no agreement; 0.01–0.20: slight agreement; 0.21–0.40: fair agreement; 0.41–0.60: moderate agreement; 0.61–0.80: substantial agreement; and 0.81–1.00: almost perfect agreement.

Statistical significance was set as *P* <0.05. All analyses were performed with R v4.2.2.

## RESULTS

### Cohort Description

**FIGURE 2:**
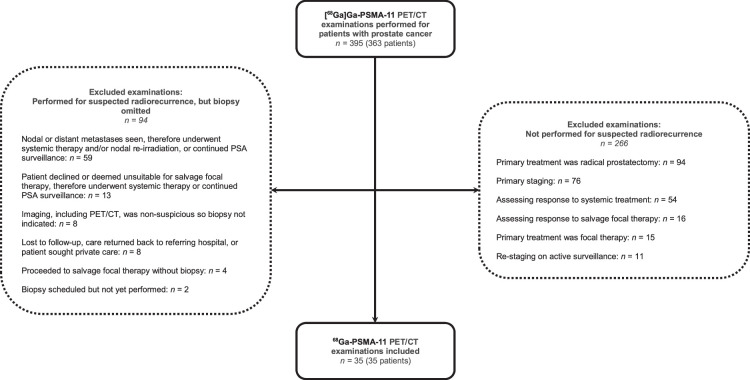
Flowchart outlining the exclusion and inclusion process of patients in this analysis.

Between 2016 and 2022, 129 [^68^Ga]Ga-PSMA-11 PET/CT scans were performed for suspected radiorecurrence, with 35 scans (35 patients) eligible for inclusion here. Figure 2 gives exclusion reasons.

Table 1 describes cohort characteristics. The majority (86%) underwent external-beam radiotherapy, with the majority (63%) using adjuvant ADT at the time of PET/CT. Median PSA at the time of PET/CT was 3.80 ng/mL [interquartile range (IQR): 2.60–5.53].

**TABLE 1:** Characteristics of Included Patients

Characteristic	n=35*
At original diagnosis	
Age (y)	63.1 (58.9, 68.3)
PSA (ng/mL)	16.9 (9.8, 34.2)
Unknown	9
Grade group	
1	7 (24)
2	9 (31)
3	5 (17)
4	5 (17)
5	3 (10)
Unknown	6
T-stage	
T1	3 (12)
T2	10 (42)
T3	11 (46)
Unknown	11
At the time of PET/CT	
Age (y)	72.4 (68.4, 75.5)
Time between diagnosis and PET/CT (y)	7.2 (5.9, 10.7)
Primary radiotherapeutic treatment	
EBRT	30 (86)
HDR brachytherapy and EBRT	2 (6)
LDR brachytherapy	3 (9)
Hormone use during primary treatment	
Adjuvant	8 (30)
Neoadjuvant	7 (26)
Neoadjuvant and adjuvant	9 (33)
Nil	3 (11)
Unknown	8
Phoenix criteria met	24 (83)
Unknown (PSA nadir not available)	6
PSA (ng/mL)	3.80 (2.60, 5.53)

* Median (IQR); n (%).

EBRT indicates external beam radiotherapy; HDR, high dose-rate; LDR, low dose-rate.

**FIGURE 3:**
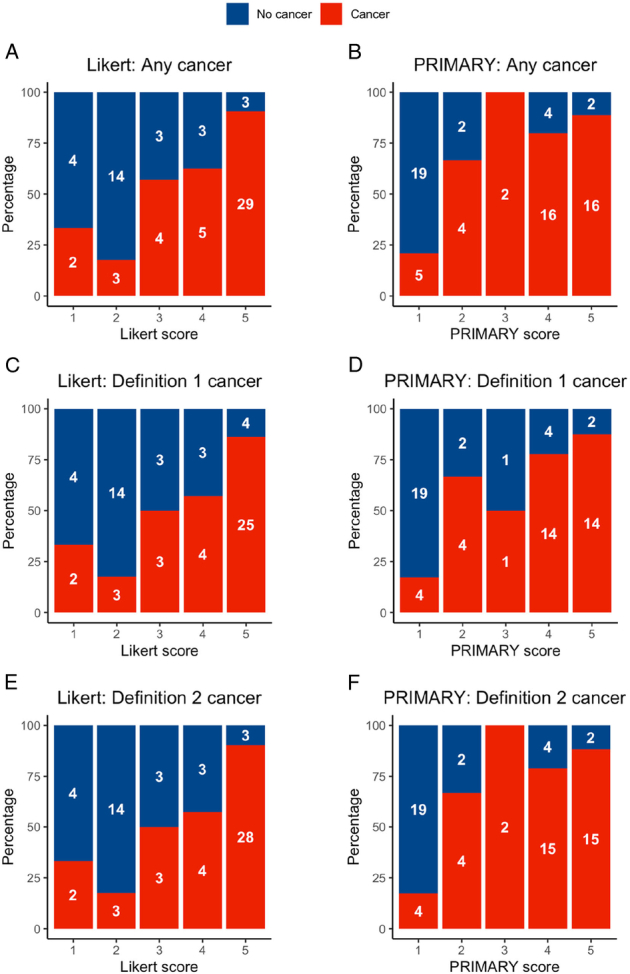
A–F, In hemi-gland analysis, stacked bar charts illustrating the proportions of cancer versus no cancer diagnosed at each score for each scoring system and for each cancer definition. The values superimposed on each bar give absolute numbers.

Forty-three out of 70 hemi-glands (61%) had cancer identified on biopsy, with PROMIS definition 1 and 2 cancer identified in 37/65 (57%) and 40/67 (60%) hemi-glands, respectively. Forty-seven out of 70 hemi-glands (67%) were assigned an E-PSMA Likert score of 3–5, with 40/70 hemi-glands (57%) assigned a PRIMARY score of 3–5. Forty out of 70 (57%) were assigned an E-PSMA Likert score of 4–5, with 38/70 (54%) assigned a PRIMARY score of 4–5. Figure 3 gives stacked bar charts illustrating the proportions of hemi-glands with cancer versus no cancer at each score and for each system.

### Diagnostic Accuracy

**FIGURE 4:**
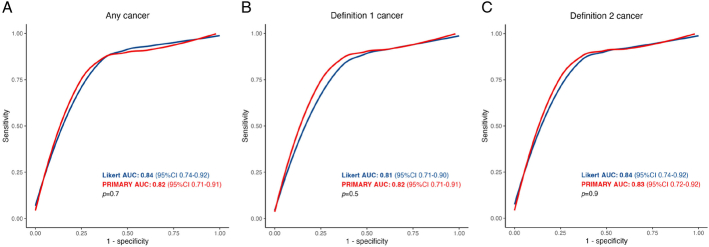
A–C, In hemi-gland analysis, smoothed cluster-bootstrapped ROC curves plotted for each cancer definition based on E-PSMA Likert and PRIMARY scores. AUC values are given for each curve.

Figure 4 illustrates the ROC curve and AUC values for each system and for each cancer definition. Supplemental Figure S1 (Supplemental Digital Content 1, http://links.lww.com/CNM/A582) gives ROC curves and AUC for each reader. For cancer of any grade or length, AUC was high for both systems but not statistically significantly different between these (E-PSMA Likert: 0.84, 95% CI: 0.74–0.92; PRIMARY: 0.82, 95% CI: 0.71–0.91; *P* = 0.7). AUC values were comparable between E-PSMA Likert and PRIMARY scores for definition 1 cancer (0.81 vs 0.82, respectively; *P* = 0.5), and for definition 2 cancer (0.84 vs 0.83, respectively; *P* = 0.9). In all cases, ROC curves showed only one major inflection point.

Table 2 details diagnostic accuracy metrics for the E-PSMA Likert and PRIMARY systems at score thresholds of both ≥3 and ≥4. Supplemental Table S1 (Supplemental Digital Content 1, http://links.lww.com/CNM/A582) gives diagnostic accuracy metrics for each reader. For diagnosis of cancer of any grade or length, sensitivity with the E-PSMA Likert system was 0.89 (95% CI: 0.78–0.96), not statistically significantly different when compared with the PRIMARY system (0.79; 95% CI: 0.67–0.89, *P* = 0.1). Specificity with the E-PSMA Likert system was 0.67 (95% CI: 0.46–0.86), not statistically significantly different when compared with the PRIMARY system (0.78; 95% CI: 0.60–0.91; *P* = 0.4). Comparable metrics were observed for both systems when investigating PROMIS definition 1 and 2 cancers (Table 2).

**TABLE 2:** In Hemi-gland Analysis, the Number of Cancers Identified Per Cancer Definition, Together With Diagnostic Accuracy Metrics for Each Scoring System

	Score ≥3 Threshold			Score ≥4 Threshold		
	E-PSMA Likert	PRIMARY	*P*	E-PSMA Likert	PRIMARY	*P*
Any cancer n=43/70						
Sensitivity	0.89 (0.78–0.96)	0.79 (0.67–0.89)	0.1	0.79 (0.67–0.90)	0.74 (0.61–0.86)	0.1
Specificity	0.67 (0.46–0.86)	0.78 (0.60–0.91)	0.4	0.78 (0.59–0.94)	0.78 (0.61–0.92)	1
PPV	0.81 (0.67–0.92)	0.85 (0.74–0.95)	0.3	0.85 (0.70–0.96)	0.84 (0.70–0.94)	0.9
NPV	0.79 (0.59–0.92)	0.69 (0.52–0.85)	0.1	0.70 (0.53–0.85)	0.66 (0.48–0.81)	0.4
PROMIS definition 1 Grade group ≥3 and/or MCCL ≥6 mm n=37/65						
Sensitivity	0.87 (0.75–0.95)	0.78 (0.66–0.89)	0.2	0.78 (0.66–0.90)	0.76 (0.61–0.88)	1
Specificity	0.65 (0.45–0.82)	0.75 (0.57–0.88)	0.4	0.75 (0.57–0.92)	0.79 (0.62–0.92)	1
PPV	0.77 (0.60–0.90)	0.81 (0.67–0.93)	0.3	0.80 (0.63–0.94)	0.82 (0.66–0.94)	0.7
NPV	0.79 (0.59–0.92)	0.72 (0.54–0.88)	0.3	0.70 (0.53–0.85)	0.71 (0.54–0.85)	0.8
PROMIS definition 2 Grade group ≥2 and/or MCCL ≥4 mm n=40/67						
Sensitivity	0.88 (0.76–0.96)	0.80 (0.68–0.90)	0.2	0.80 (0.68–0.91)	0.75 (0.61–0.87)	0.6
Specificity	0.67 (0.46–0.86)	0.78 (0.60–0.91)	0.4	0.78 (0.59–0.94)	0.78 (0.61–0.92)	1
PPV	0.80 (0.64–0.92)	0.84 (0.71–0.95)	0.3	0.84 (0.69–0.96)	0.83 (0.68–0.94)	0.8
NPV	0.79 (0.59–0.92)	0.72 (0.54–0.88)	0.3	0.70 (0.53–0.85)	0.68 (0.51–0.82)	0.4

When the score threshold for a suspicious examination increased from ≥3 to ≥4, diagnostic performance for both systems remained similar. For diagnosis of cancer of any grade or length, sensitivity with the E-PSMA Likert system reduced to 0.79 (95% CI: 0.67–0.90), comparable to the PRIMARY system (0.74; 95% CI: 0.61–0.86; *P* = 0.1). Specificity of the E-PSMA Likert system increased to 0.78 (95% CI: 0.59–0.94), again comparable to the PRIMARY system (0.78; 95% CI: 0.61–0.92*; P* = 1). PPV was also similar between E-PSMA Likert (0.85; 95% CI: 0.70–0.96) and PRIMARY systems (0.84; 95% CI: 0.70–0.94; *P* = 0.9). Last, NPV was comparable (E-PSMA Likert: 0.70, 95% CI: 0.53–0.85; PRIMARY: 0.66, 95% CI: 0.48–0.81; *P* = 0.4). As with a score threshold ≥3, diagnostic metrics were comparable when the definition of cancer was altered to PROMIS definitions 1 and 2 (Table 2).

### Interreader Agreement

For categorization of scores into 1–2 versus 3–5, there was substantial interreader agreement between the 2 readers for both E-PSMA Likert and PRIMARY systems (E-PSMA Likert: κ = 0.65; 95% CI: 0.44–0.83; PRIMARY: κ = 0.74; 95% CI: 0.59–0.90). There also remained substantial interreader agreement for both systems when categorizing scores into 1–3 versus 4–5 (E-PSMA Likert: κ = 0.72; 95% CI: 0.55–0.88; PRIMARY: κ = 0.74; 95% CI: 0.59–0.90).

### Whole Gland Analysis

Of the patients, 28/35 (80%) had cancer identified on biopsy; 24/32 (75%) and 27/34 (79%) had PROMIS definition 1 and 2 cancer, respectively. Of the patients, 31/35 (89%) were given a E-PSMA Likert score of 3–5, compared with 28/35 (80%) given a PRIMARY score of 3–5. In contrast, 28/35 patients (80%) were given a E-PSMA Likert score of 4–5, compared with 26/35 (74%) given a PRIMARY score of 4–5. Supplemental Figure S2 (Supplemental Digital Content 1, http://links.lww.com/CNM/A582) gives stacked bar charts illustrating the proportions of patients with cancer versus no cancer at each score and for each system.

Supplemental Figure S3 (Supplemental Digital Content 1, http://links.lww.com/CNM/A582) illustrates ROC curves and AUC values for the E-PSMA Likert and PRIMARY systems at the whole gland level. AUC for the E-PSMA Likert system ranged from 0.72 to 0.78 across cancer definitions, compared with 0.81–0.83 for the PRIMARY system. No comparison of AUC was statistically significant. Supplemental Table S2 (Supplemental Digital Content 1, http://links.lww.com/CNM/A582) details the diagnostic accuracy metrics for these systems. At a score threshold of ≥3, sensitivity was very high for both E-PSMA Likert systems (E-PSMA Likert: 0.96 for each cancer definition; PRIMARY: 0.92–0.93). Point estimates for specificity were lower for the E-PSMA Likert system (0.38–0.43) versus PRIMARY (0.62–0.71). PPV and NPV were comparable between systems. In this small whole gland cohort, no comparison for sensitivity, specificity, PPV, or NPV was statistically significant.

For categorization of scores into 1–2 versus 3–5, there was moderate interreader agreement between the 2 readers for the E-PSMA Likert system (κ = 0.60; 95% CI: 0.25–0.95), but almost perfect agreement for the PRIMARY system (κ = 0.84; 95% CI: 0.62–1.00). For categorization of scores into 1–3 versus 4–5, interreader agreement for the E-PSMA Likert system improved to substantial (κ = 0.77; 95% CI: 0.52–1.00), while agreement for the PRIMARY system remained almost perfect (κ = 0.86; 95% CI: 0.67–1.00).

## DISCUSSION

### Summary

Our results demonstrate that the 5-point PRIMARY scoring system has high diagnostic accuracy with substantial interreader agreement for the detection of localized radiorecurrent prostate cancer at both hemi-gland and whole gland levels using [^68^Ga]Ga-PSMA-11 PET/CT. However, the PRIMARY score did not demonstrate improved performance when compared with the 5-point E-PSMA Likert score when applied by expert readers. It should be noted that the E-PSMA Likert system demonstrated higher sensitivity and lower specificity point estimates for the detection of all 3 cancer types, although these differences were not statistically significantly different in this small study. Also, on visualization of ROC curves, there was consistently only one major inflection point. This suggests 5-point scoring systems for both PRIMARY and Likert could be redundant in this setting, with a simpler 3-point version providing comparable performance.

### Context Within the Literature

Few studies have examined the role of PSMA PET/CT in the detection of intraprostatic radiorecurrent cancer, and only 4 have validated findings histologically.^15^ Of these, only patients with a positive PET/CT were biopsied, and PET/CT interpretation criteria were not consistent. Pfister and colleagues retrospectively reported on 50 patients who had undergone [^68^Ga]Ga-PSMA-11 PET/CT before salvage radical prostatectomy. PET/CT interpretation criteria were not stated, but sensitivity and specificity on a hemi-gland level were 81% and 67% respectively, with a sensitivity of 100% at the whole gland level.^17^ Raveenthiran et al^18^ retrospectively reported on 267 patients undergoing [^68^Ga]Ga-PSMA-11 PET/CT; 157/267 patients (57%) had suspicious uptake in the prostate, defined as a moderate or intense PSMA-avid lesion with SUV_max_ ≥3.0. Thirty-three patients underwent biopsy, yielding a sensitivity of 85%. Song et al^19^ published a prospective series of [^18^F]DCFPyL PET/CT in 30 patients, half of whom had suspicious prostatic uptake defined as “uptake above background.” Three patients underwent prostate biopsy, yielding a sensitivity of 100%. Last, Rasing et al^20^ published a prospective series of 41 patients undergoing [^68^Ga]Ga-PSMA-11 PET/CT with MRI and biopsy. All 41 had a positive PET/CT defined as “focal increased uptake.” PET/CT-specific diagnostic accuracy was not presented, but sensitivity aggregating PET/CT and MRI data together was 100%. Although these studies are limited by their single-centre, single-arm nature, and only biopsy patients with positive imaging, there is certainly a signal that PSMA PET/CT has value in identifying intraprostatic radiorecurrent disease, as has been shown in the primary diagnostic setting.^23^ Methods to optimize its use, for example, creation of effective scoring systems, should be a high priority given the poor outcomes of patients with radiorecurrence, and given the emergence of local salvage treatments that can improve this.^3,6,7^ To our knowledge, this is the only study evaluating the PRIMARY score in the radiorecurrent setting.

### Structured Versus Likert Scoring Systems

Initially, an adapted 5-point Likert score was recommended by E-PSMA guidelines for assessing [^68^Ga]Ga-PSMA-11 PET/CT findings regardless of anatomic location.^22^ Subsequently, the PROMISE v2 framework incorporated the PRIMARY system for assessment of intraprostatic disease.^26^ In the untreated prostate, the PRIMARY system has high sensitivity for detecting clinically-significant cancer, with substantial interreader agreement between expert readers that exceeds that of the MRI PI-RADS system.^23–25^ Before these studies, interreader agreement for PSMA PET/CT has only been assessed for TNM staging and not for localization of intraprostatic disease.^35–37^ Our study is the first to assess interreader agreement for intraprostatic disease in the radiorecurrent setting, where we have shown substantial interreader agreement between two expert readers for both PRIMARY and E-PSMA Likert systems.

Although no specific criteria are given, Likert systems are popular radiologic scoring systems owing to being flexible and intuitive. For cancer detection in untreated prostates, a 5-point MRI Likert system has comparable diagnostic accuracy to the PI-RADS system, and is currently preferred by UK guidelines.^38–44^ Our group has also previously shown a 5-point Likert score to have high sensitivity for MRI detection of radiorecurrent disease in a prospective trial.^7^ However, as Likert systems lack specific interpretation criteria, their utility is arguably reduced in less-experienced readers.^40,42^ In a previous prospective multireader study of 40 [^68^Ga]Ga-PSMA-11 PET/CT scans in the biochemical failure setting postradiotherapy or postprostatectomy, interreader agreement for T-staging was fair between low-experience readers (κ = 0.35), moderate (κ = 0.55) between intermediate-experience readers, and was only substantial for high-experience readers.^45^ It has been previously highlighted that, postradiotherapy, faint-to-moderate prostatic uptake and inflammation comprise the majority of false-positive reads, while adjacent urine activity can cause false-negative reads.^46^ A structured scoring system with specific criteria like PRIMARY could therefore be advantageous with less-experienced readers.

Scoring systems like PRIMARY can also be adapted to the posttreatment setting and iteratively improved as validation data emerge, which may further improve diagnostic accuracy. First, scoring criteria could be adapted to take account of the primary site of cancer preradiotherapy as established through previous imaging. This anatomic information is a major focus of the recently published PI-RR MRI scoring system for detecting radiorecurrent disease.^47^ Second, further SUV_max_ cutoffs could be incorporated. Although an SUV_max_ >12 denotes a maximum PRIMARY score of 5, we have previously shown that lower values have good diagnostic value in the radiorecurrent setting. For example, using an SUV_max_ cutoff of 4.4 yields a sensitivity of 86%, specificity 84%, PPV 85%, and NPV 78% for detecting cancer of any grade or length.^21^ A lower SUV_max_ cutoff than 12 could, therefore, be useful in scoring more indeterminate cases, at least within the radiorecurrent setting. The ROC curves generated in our analyses also suggest a simpler 3-point version of both PRIMARY and Likert could be sufficient in this radiorecurrent setting in place of a more complex 5-point version.

### Future Research

Further validation of the PRIMARY score in the radiorecurrent setting is warranted, which may in turn lead to beneficial modifications to scoring criteria as described previously. Validation studies should ideally occur within a prospective setting with uniform indications and protocols for biopsy, as has been conducted with MRI postradiotherapy.^7^ A strength of this work is that patients with negative PET/CT have been included, with the use of hemi-gland analysis further increasing the number of negative units of analysis. It is noted that previous studies in this field have only biopsied patients with positive imaging, which significantly biases results.^17–20^ It is possible that the PRIMARY system could have better specificity than the Likert system, but this needs to be demonstrated clearly in patients with negative imaging. Such studies should be coupled with investigations into interreader agreement, particularly regarding less experienced readers, as discussed. On a related note, how PRIMARY scores and other radiologic data like SUV_max_ correlate with outcomes after local salvage treatment should be established, and these may be a useful addition to existing predictive risk models in this setting.^48–50^ Last, evaluation of how PRIMARY scores adds value to MRI diagnostics is needed. Prostate MRI in this same cohort has previously been shown to have sensitivity of 0.72–0.76, specificity 0.64–0.65, PPV 0.74–0.76, and NPV 0.59–0.67 depending on cancer definition used.^21^ When prostate MRI and [^68^Ga]Ga-PSMA-11 PET/CT, scored using a Likert system, were interpreted in conjunction, there were statistically significant increases in sensitivity to 0.97–0.98 and in NPV to 0.93. Interpretation of MRI using a dedicated scoring system like PI-RR could also add value.^47^ In the future, it may even be that a combined and dedicated postradiotherapy PSMA PET/CT and multiparametric MRI scoring system proves most valuable.

### Limitations

This study is subject to limitations. This is a single-centre retrospective series, with its biggest limitation being cohort size. However, this cohort does represent referrals from elsewhere in the UK, both for [^68^Ga]Ga-PSMA-11 PET/CT and salvage focal therapy. Ultimately, our cohort size primarily reflects the paucity of patients undergoing biopsy in this setting, and this corresponds with other series evaluating both [^68^Ga]Ga-PSMA-11 PET/CT and salvage focal therapy.^11,17–20^ To reach biopsy, this implies a given patient is fit both for biopsy and subsequent local salvage therapy (here at a median age of over 72 y and a median of over 7 y since completing radiotherapy), and has no extraprostatic disease identified that would contraindicate local salvage.^13^

In addition, although we have included 4 patients with negative imaging and although we have used a hemi-gland analysis to increase negative units of analysis, 8 patients were excluded as they did not proceed to prostate biopsy, as they were deemed to have negative imaging. The potential heterogeneity in clinical practice is a limitation of the retrospective design. Furthermore, focusing on a biopsied cohort with predominantly positive PET/CT imaging does introduce partial verification bias.^51^ This is known to inflate sensitivity and deflate specificity estimates. This study also uses 2 expert readers, and we are, therefore, unable to establish how useful these scoring systems would be for less-experienced readers.

Hemi-gland data are also nonindependent, although we have mitigated against this by use of cluster bootstrapping. Lastly, biopsy protocols were not uniform, which reflects a limitation of retrospective data. However, most biopsies were transperineal and all included comprehensive bilateral sampling, including both peripheral zones, which is sufficient for focal therapy planning.

## CONCLUSIONS

In patients with biochemical recurrence after primary radiotherapy for prostate cancer, both the 5-point PRIMARY and E-PSMA Likert scoring systems score demonstrated good performance and substantial interreader agreement between experts for detection of intraprostatic recurrences.

## Supplementary Material
