# Supplementary material for: Validation of the PSMA PRIMARY Scoring System and Comparison to an E-PSMA Likert System for [68Ga]Ga-PSMA-11 PET/CT Interpretation in Men With Suspected Radiorecurrent Prostate Cancer
Source: Clin Nucl Med. 2025 Nov 27;51(1):e1–e10. doi: 10.1097/RLU.0000000000006168 (PMC12673892; doi:10.1097/RLU.0000000000006168)
Supplement: Supplementary file 1 [file rlu-51-e1-s001.docx]

**SUPPLEMENTARY MATERIAL**

**
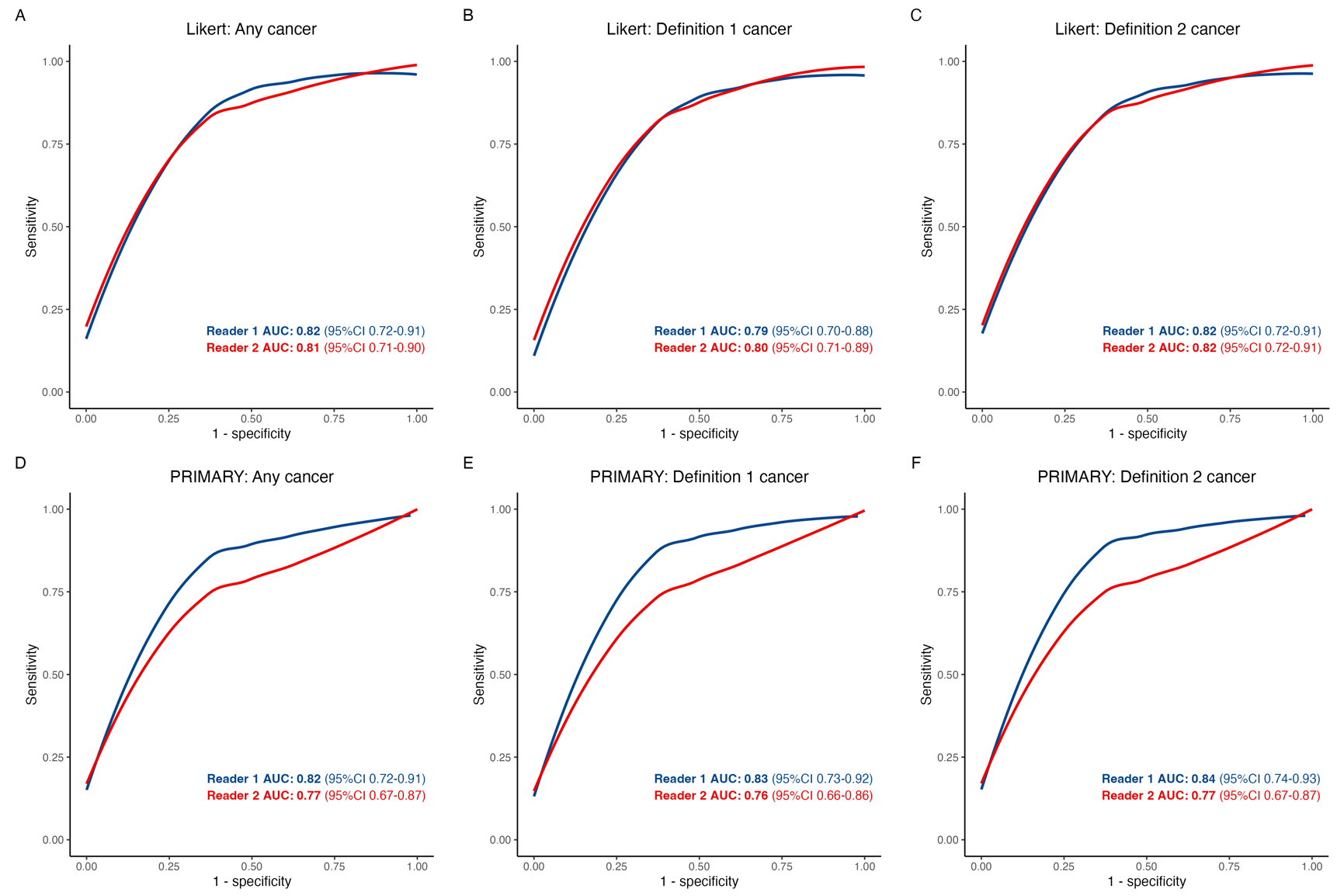
Fig.S1:** In hemi-gland analysis, smoothed cluster-bootstrapped ROC curves plotted for each reader for each scoring system and for each cancer definition. AUC values are given for each curve.

|  | **Score ≥3 threshold** | | | | **Score ≥4 threshold** | | | |
| --- | --- | --- | --- | --- | --- | --- | --- | --- |
|  | **E-PSMA Likert**  **Reader 1** | **E-PSMA Likert**  **Reader 2** | **PRIMARY**  **Reader 1** | **PRIMARY**  **Reader 2** | **E-PSMA Likert**  **Reader 1** | **E-PSMA Likert**  **Reader 2** | **PRIMARY**  **Reader 1** | **PRIMARY**  **Reader 2** |
| **Any cancer**  *n* = 43/70 | | |  |  |  |  |  |  |
| Sensitivity | 0.86 (0.76-0.95) | 0.74 (0.63-0.85) | 0.74 (0.63-0.87) | 0.67 (0.57-0.79) | 0.74 (0.63-0.87) | 0.67 (0.56-0.79) | 0.70 (0.57-0.83) | 0.63 (0.51-0.74) |
| Specificity | 0.70 (0.52-0.87) | 0.82 (0.67-0.95) | 0.81 (0.67-0.94) | 0.82 (0.67-0.95) | 0.82 (0.64-0.96) | 0.86 (0.71-0.97) | 0.81 (0.67-0.94) | 0.82 (0.67-0.95) |
| PPV | 0.83 (0.69-0.93) | 0.87 (0.74-0.97) | 0.87 (0.75-0.96) | 0.86 (0.72-0.96) | 0.87 (0.72-0.97) | 0.89 (0.77-0.97) | 0.86 (0.73-0.95) | 0.85 (0.70-0.96) |
| NPV | 0.77 (0.58-0.91) | 0.67 (0.48-0.82) | 0.67 (0.49-0.83) | 0.62 (0.44-0.76) | 0.67 (0.51-0.82) | 0.62 (0.46-0.77) | 0.62 (0.47-0.79) | 0.58 (0.42-0.73) |
| **PROMIS definition 1**  Grade group ≥3 and/or MCCL ≥6mm  *n* = 37/65 | | |  |  |  |  |  |  |
| Sensitivity | 0.84 (0.72-0.94) | 0.73 (0.60-0.85) | 0.76 (0.62-0.88) | 0.65 (0.53-0.77) | 0.76 (0.62-0.88) | 0.65 (0.53-0.78) | 0.73 (0.59-0.86) | 0.62 (0.50-0.75) |
| Specificity | 0.68 (0.50-0.84) | 0.79 (0.64-0.92) | 0.79 (0.64-0.93) | 0.79 (0.64-0.92) | 0.79 (0.61-0.94) | 0.83 (0.69-0.96) | 0.82 (0.67-0.94) | 0.83 (0.67-0.95) |
| PPV | 0.78 (0.62-0.90) | 0.82 (0.66-0.94) | 0.83 (0.69-0.94) | 0.81 (0.64-0.93) | 0.83 (0.68-0.97) | 0.83 (0.69-0.96) | 0.85 (0.71-0.95) | 0.83 (0.66-0.95) |
| NPV | 0.77 (0.58-0.91) | 0.69 (0.52-0.85) | 0.71 (0.54-0.87) | 0.63 (0.46-0.78) | 0.67 (0.51-0.82) | 0.64 (0.48-0.79) | 0.69 (0.54-0.85) | 0.62 (0.45-0.77) |
| **PROMIS definition 2**  Grade group ≥2 and/or MCCL ≥4mm  *n* = 40/67 | | |  |  |  |  |  |  |
| Sensitivity | 0.85 (0.74-0.95) | 0.75 (0.64-0.86) | 0.78 (0.65-0.89) | 0.68 (0.56-0.80) | 0.78 (0.65-0.89) | 0.68 (0.56-0.80) | 0.73 (0.61-0.85) | 0.63 (0.51-0.75) |
| Specificity | 0.70 (0.52-0.87) | 0.82 (0.67-0.86) | 0.81 (0.67-0.94) | 0.82 (0.67-0.95) | 0.82 (0.64-0.96) | 0.86 (0.71-0.97) | 0.81 (0.67-0.94) | 0.82 (0.67-0.95) |
| PPV | 0.82 (0.67-0.93) | 0.86 (0.72-0.97) | 0.86 (0.75-0.96) | 0.85 (0.70-0.96) | 0.86 (0.72-0.97) | 0.88 (0.75-0.97) | 0.86 (0.73-0.95) | 0.84 (0.68-0.96) |
| NPV | 0.77 (0.58-0.91) | 0.69 (0.52-0.85) | 0.71 (0.54-0.87) | 0.63 (0.46-0.78) | 0.67 (0.51-0.82) | 0.64 (0.48-0.79) | 0.67 (0.50-0.82) | 0.59 (0.44-0.74) |

**Table S1:** In hemi-gland analysis, the number of cancers identified per cancer definition, together with diagnostic accuracy metrics for each reader for each scoring system.

**Fig.S2:** In whole gland analysis, stacked bar charts illustrating the proportions of cancer versus no cancer diagnosed at each score for each scoring system and for each cancer definition. The values superimposed on each bar give absolute numbers.**
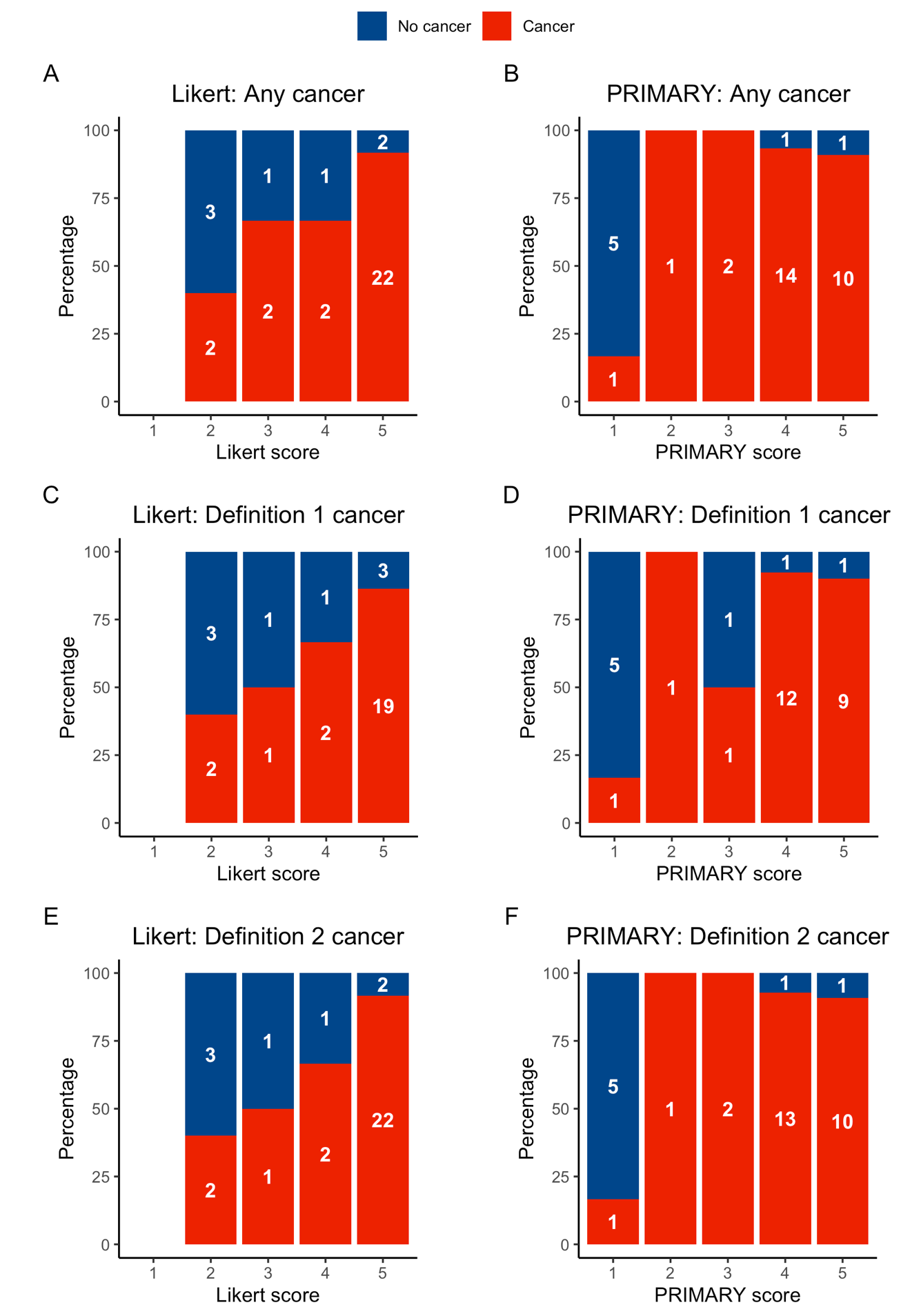
**


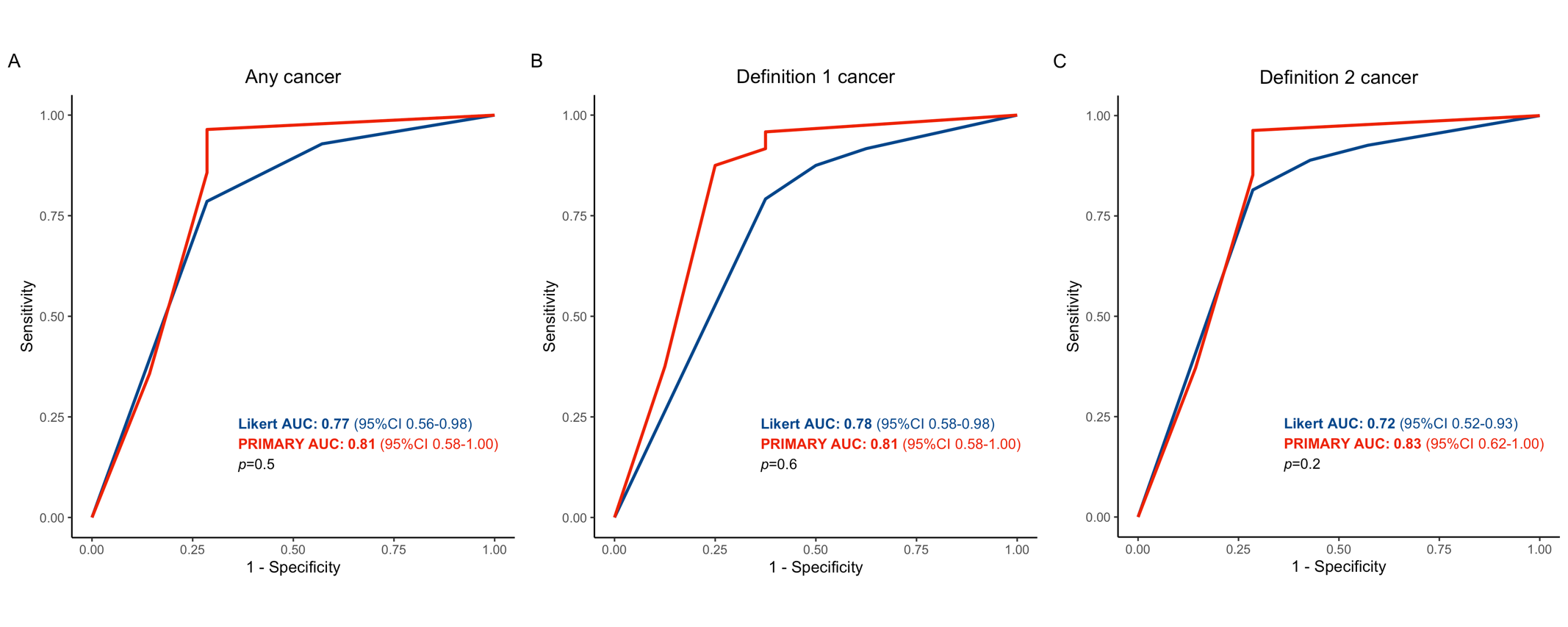
**Fig.S3:** In whole-gland analysis, ROC curves plotted for each cancer definition based on E-PSMA Likert and PRIMARY scores. AUC values are given for each curve.

|  | **Score ≥3 threshold** | | | **Score ≥4 threshold** | | | |
| --- | --- | --- | --- | --- | --- | --- | --- |
|  | **E-PSMA Likert** | **PRIMARY** | ***p*** | | **E-PSMA Likert** | **PRIMARY** | ***p*** |
| **Any cancer**  *n* = 28/35 | | | | | | | |
| Sensitivity | 0.96 (0.82-1.00) | 0.93 (0.76-0.99) | 1 | | 0.89 (0.72-0.98) | 0.86 (0.67-0.96) | 1 |
| Specificity | 0.43 (0.10-0.82) | 0.71 (0.29-0.96) | 0.5 | | 0.57 (0.18-0.90) | 0.71 (0.29-0.96) | 1 |
| PPV | 0.87 (0.70-0.96) | 0.93 (0.76-0.99) | 0.2 | | 0.89 (0.72-0.98) | 0.92 (0.75-0.99) | 0.4 |
| NPV | 0.75 (0.19-0.99) | 0.71 (0.29-0.96) | 0.8 | | 0.57 (0.18-0.90) | 0.56 (0.21-0.86) | 0.9 |
| **PROMIS definition 1**  Grade group ≥3 and/or MCCL ≥6mm  *n* = 24/32 | | | | | | | |
| Sensitivity | 0.96 (0.79-1.00) | 0.92 (0.73-0.99) | 1 | | 0.88 (0.72-0.98) | 0.88 (0.68-0.97) | 1 |
| Specificity | 0.38 (0.09-0.76) | 0.62 (0.24-0.91) | 0.5 | | 0.57 (0.18-0.90) | 0.75 (0.35-0.97) | 0.5 |
| PPV | 0.82 (0.63-0.94) | 0.88 (0.69-0.97) | 0.2 | | 0.89 (0.72-0.98) | 0.91 (0.72-0.99) | 0.1 |
| NPV | 0.75 (0.19-0.99) | 0.71 (0.29-0.96) | 0.8 | | 0.57 (0.18-0.90) | 0.67 (0.30-0.93) | 0.5 |
| **PROMIS definition 2**  Grade group ≥2 and/or MCCL ≥4mm  *n* = 27/34 | | | | | | | |
| Sensitivity | 0.96 (0.81-1.00) | 0.93 (0.76-0.99) | 1 | | 0.89 (0.71-0.98) | 0.85 (0.66-0.96) | 1 |
| Specificity | 0.43 (0.10-0.82) | 0.71 (0.29-0.96) | 0.5 | | 0.57 (0.18-0.90) | 0.71 (0.29-0.96) | 1 |
| PPV | 0.87 (0.69-0.96) | 0.93 (0.76-0.99) | 0.2 | | 0.89 (0.71-0.98) | 0.92 (0.74-0.99) | 0.4 |
| NPV | 0.75 (0.19-0.99) | 0.71 (0.29-0.96) | 0.8 | | 0.57 (0.18-0.90) | 0.56 (0.21-0.86) | 0.9 |

**Table S2:** In whole gland analysis, the number of cancers identified per cancer definition, together with diagnostic accuracy metrics for each scoring system.
